# Initialization of latent space coordinates *via* random linear projections for learning robotic sensory-motor sequences

**DOI:** 10.3389/fnbot.2022.891031

**Published:** 2022-09-14

**Authors:** Vsevolod Nikulin, Jun Tani

**Affiliations:** Cognitive Neurorobotics Research Unit, Okinawa Institute of Science and Technology, Onna, Japan

**Keywords:** generative models, robotics, latent encoding, random projection, motion primitives, Recurrent Neural Network

## Abstract

Robot kinematic data, despite being high-dimensional, is highly correlated, especially when considering motions grouped in certain primitives. These almost linear correlations within primitives allow us to interpret motions as points drawn close to a union of low-dimensional affine subspaces in the space of all motions. Motivated by results of embedding theory, in particular, generalizations of the Whitney embedding theorem, we show that random linear projection of motor sequences into low-dimensional space loses very little information about the structure of kinematic data. Projected points offer good initial estimates for values of latent variables in a generative model of robot sensory-motor behavior primitives. We conducted a series of experiments in which we trained a Recurrent Neural Network to generate sensory-motor sequences for a robotic manipulator with 9 degrees of freedom. Experimental results demonstrate substantial improvement in generalization abilities for unobserved samples during initialization of latent variables with a random linear projection of motor data over initialization with zero or random values. Moreover, latent space is well-structured such that samples belonging to different primitives are well separated from the onset of the training process.

## 1. Introduction

Generative models allow representation of high-dimensional behavior patterns (sequences of action-perception pairs) in much lower-dimensional latent space. However, these representations are far from unique. It is convenient for analysis and theoretical discussions of generalization capabilities if encoded points exhibit some regularity. This paper focuses on the issue of efficient encoding of motion primitives in generative models for robotic behavior. Such models are designed to select robots actions *via* generating predictions of them. A search in latent space is conducted to generate desired motion. Thus, it is advantageous to have well-structured latent space.

PCA analysis of human movement suggests that most of the variance is explained by only a few components (Sanger, 2000), which confirms an idea of the pioneer of kinesiology, Nikolai Bernstein. Bernstein (1996) proposed that human motion, despite being high-dimensional can be described using low-dimensional points. In other words, motion primitives can be represented in a compact manner using a small number of variables to encode flexible adaptations to variations of related features, such as positions of target objects, size and shape of objects, initial position of a manipulator, etc.

Motion primitives are an indispensable concept both in robotic and human behavior modeling. They provide modularity in construction of complex interactions with an environment. Primitives are considered minimal sets of reusable patterns to be combined to generate diverse patterns. The importance of motion primitives for robotic behavior design is discussed in Schaal (1999). In their work, the authors specify two types of motion primitives: discrete and cyclic, which correspond to fixed points and limit cycles in dynamic systems, respectively. Each of them can be represented by a single point in the parameter space of dynamic systems.

There are many approaches to learning behavioral or motion primitives. One is described in Schaal et al. (2004): direct modeling differential equations for discrete and oscillating patterns with variable parameters tuned by reinforcement learning. A similar approach is taken in Ude et al. (2010) with the emphasis on two types of primitives. However, the training is goal-oriented in both cases. The reward function is designed to ensure specific dynamic properties of a trajectory and to ensure reaching a certain final state. In reality, however, there are many constraints regarding interactions with objects in an environment in a certain way, which are hard to take into account when hand-designed reward functions are used. It is theoretically possible to extract those constraints automatically *via* supervised learning through imitation of recorded trajectories, if data are plentiful. For example, in Noda et al. (2014) an autoencoder is used to create a generative model for multimodal primitives.

We consider a supervised learning scenario in which every motion has finite encoding and can be regenerated using this encoding and a shared generative model implemented as a Recurrent Neural Network. In our previous work (Nikulin and Tani, 2020), we demonstrated that explicit embedding of hypersurfaces corresponding to each motion primitive in shared latent space enhances inter-primitive generalization capacity. However, this approach requires manual labeling of learning data, which may be infeasible when dealing with large datasets. In this paper, we address the issue of finding suitable latent representations of sensory-motor data, which can be automatically clustered for each corresponding primitive.

The first thing to notice is the high correlation between motor and sensory (typically visual) information. Information contained in a sequence of joint angles of a robotic manipulator allows us to partially generate a visual sequence. For example, the manipulator is reaching for an object and then pushing it in some direction. A sequence of joint angles in this movement provides information about the location of the object at any moment in time. Therefore, by having appropriate encoding of kinematic data we can reconstruct sensory information with some precision. From these considerations, we assume that kinematic data can be used to initialize values of latent variables for each sample prior to learning weights of the RNN for a generative model.

Clustering motion primitives is an intricate problem. Motions corresponding to different primitives can be located very close to each other in the space of all sequences of joint angles, which is referred to as “trajectory space.” For example, let us consider cases of a robotic manipulator reaching for and grasping or reaching for and simply touching an object in the same location in space. Both sequences are examples of different primitives, yet located close to each other in the space of all sequences. This shows that utilizing classic distance-based clustering schemes can potentially yield poor results. However, motor sequences belonging to specific primitives can be efficiently encoded by a very small number of variables, e.g., position of the object in space, thus forming low-dimensional manifolds in trajectory space. The main assumption we are making is the following: the aforementioned manifolds lie inside independent, low-dimensional, affine subspaces, i.e., sequences belonging to the same primitive can be expressed as a sparse affine combination of one another. This enables us to use the affine subspace clustering algorithm, which shows good results in our experiments.

However, even if we drop the assumption about linearity, we can linearly project low-dimensional manifolds from trajectory space into parameter space without overlapping primitives. In Calinon et al. (2007), the authors use projection of trajectory data into dominant principal components for further probabilistic modeling. A corollary of the Whitney embedding theorem, described in Sauer et al. (1991), tells us that *almost all* linear projections will have the required property; thus, a random linear projection will suffice. Moreover, we show that random projection is also robust enough if the parameter space has sufficient dimensions.

Here we test two hypotheses: (i) motion primitives lie inside independent, affine subspaces in trajectory space, and (ii) random linear projection of motion data to latent space and consecutive learning of the generative model together with latent representations keep linear manifolds at a degree sufficient for subspace clustering, which ensures robustness and separation of encoding for different primitives. To test the first hypothesis, we analyse generated joint-angle sequences of a humanoid robot obtained *via* mapping of motion-capture data. To test the second hypothesis, we design artificial data with a sufficient number of trajectories to test the generalization capacity of the model. Two types of generalization are tested: intra- and inter-primitive generalizations. The former is the ability of the model to generate unseen samples that belong to the learned primitives and the latter is the ability to accommodate new primitives that must be learned. Notice, it is not zero-shot or one-shot learning.

## 2. Background

### 2.1. Generative models and predictive coding

According to predictive coding theory, formulated in Rao and Ballard (1999) and Friston et al. (2006), behavior of an agent, e.g., a robotic manipulator, can be modeled as constant generation of predicted sensory information **o**_*t*_ based on some changing internal state **d**_*t*_ at any moment in time *t*. Sensory information includes proprioception, which are joint angle positions that can be used to determine which commands should be sent in order for a robot to satisfy predicted future positions of manipulators. We split information **o**_*t*_ into two parts: proprioception **m**_*t*_, which we will refer to as “motor commands” for the sake of brevity, and the rest of the perception **s**_*t*_. Predicted information is compared with actual information and the internal state is corrected based on the error. Internal state dynamics can be modeled as a deterministic process using RNN (Annabi et al., 2021), for instance MTRNN (Yamashita and Tani, 2008), LSTM (Graves, 2014), or as a stochastic process, such as PVRNN (Ahmadi and Tani, 2019). Correction of the entire internal state is computationally costly due to its high dimension. Therefore, a sequence of internal states is encoded in either a sequence of latent vectors **z**_*t*_ or a single latent vector **z**. The former is a case of PVRNN. In this study we concentrate on the latter, since we work with simple motion primitives that can be encoded as points of a finite-dimensional space.

Overall dynamics of the model of interest are described in the following equations:


(1a) $$\begin{array}{l} {\text{d}}_{0}=g\left(\text{z}\right) \end{array}$$

(1b) $$\begin{array}{l} {\text{d}}_{t+1}=f\left({\text{d}}_{t},\text{z}\right) \end{array}$$

(1c) $$\begin{array}{l} {\text{s}}_{t}=s\left({\text{d}}_{t}\right) \end{array}$$

(1d) $$\begin{array}{l} {\text{m}}_{t}=m\left({\text{d}}_{t}\right) \end{array}$$

Here **z** determines the initial state and also controls the state transition. A variable that controls the state transition is called *Parametric Bias* and is typically independent from the initial state, as in Tani and Ito (2003). In this study, we unify all information that describes a sensory-motor sequence in a single vector **z**. A detailed description of the utilized model will be provided in later sections.

### 2.2. Random linear projections

An important corollary of the Whitney Embedding Theorem, described in Sauer et al. (1991), states the following:

**Theorem 1**. Let *A* be a compact subset of ℝ^*k*^, lower boxdim(*A*) = *d*. If *q* > 2*d*, then almost every linear transformation of ℝ^*k*^ to ℝ^*q*^ is one-to-one on A.

Where lower boxdim(*A*) is a lower box dimension of *A*. In particular, if *A* is a smooth manifold, compactly restricted in some finite volume, the lower box dimension will coincide with the box-counting dimension and the regular manifold dimension. Moreover, if *A* is a union of smooth manifolds, then its box-counting dimension is equal to the maximal dimension of a manifold in the union. In other words, if some high-dimensional data lie close to a union of at most *d*-dimensional manifolds, then *n* > 2*d* random observations are sufficient to encode the data without ambiguity.

This is a side result of the paper. The main statement the authors are making is about general smooth maps instead of linear maps. The space of all smooth maps is infinitely dimensional and there is no Lebesgue measure on such a space. Therefore, the authors propose their own definition of the term “almost all” in terms of prevalence. However, this definition is not required here, and the term “almost all” is used in the usual, measure-theoretic way. The space of all linear maps is finite-dimensional. We refer the interested reader to the original literature cited above.

Let *p* be a number of variables in a single motor command for a robotic manipulator, e.g., joint angle values in the case of a PID controller. We regard a sequence of *T* motor commands **m**_1:*T*_ corresponding to a single motion as a point in ℝ^*pT*^. The main assumption of this paper is that specific motions in motion primitives are specified by a small number of variables. For example, all motions corresponding to a robotic manipulator touching an object can be encoded in just the position of the touch point, given that the initial position of the manipulator is fixed. In theory, dexterous robotic systems have many degrees of freedom and can reach the same point in potentially infinite number of ways. However, as we mentioned in introduction, there are many intrinsic constraints present in a dataset which narrow possible trajectories down to finite number of dimensions or even a single trajectory. At worst there is a finite number of additional dimensions to take into account in a set of motions belonging to one primitive. This means that all these motions are points on a smooth, low-dimensional manifold in ℝ^*pT*^, and all possible motions are described as points on a union of such primitives. Since values for motor commands are restricted by their natures, we can ensure that all available points are bounded in a finite volume, so this volume together with its boundary is compact. The above theorem guarantees that random linear transformation of ℝ^*pT*^ to ℝ^*q*^, where *q* is the dimension of latent representations, is one-to-one for all available motions given that *q* is sufficiently high.

### 2.3. Linear subspace clustering

The embedding theorem from the previous section guarantees the existence of an encoding, but it does not provide any assurance about the structure of the encoded information. In this section, we explore one assumption regarding organization of motor data, that manifolds corresponding to motion primitives are close to linear. This enables us to apply the *Subspace Clustering* algorithm described in Vidal and Favaro (2014).

The problem of subspace clustering is stated as follows: given a data matrix *Z* = [**z**_1_, **z**_2_, …, **z**_*n*_] find correct labels *l*(*i*) for each **z**_*i*_ under assumption **z**_*i*_ = Φ_*l*(*i*)_**ξ**_*i*_ + **ϵ**_*i*_, where *l*(*i*) ∈ {1, 2, …, *K*} and Φ_*l*(*i*)_ are some linear projections from low-dimensional spaces. In other words, we assume that data points **z**_*i*_ belong to the union of *K* low-dimensional subspaces with errors **ϵ**_*i*_.

#### 2.3.1. ϵ_*i*_ = 0

We start the investigation of this problem with a simplified case in which **z**_*i*_ belongs to subspaces with no error. There are many approaches to solve this problem. In this paper we concentrate on cases based on self-representation matrices. Matrix *C* is called *self-representation matrix* when *Z* = *ZC*. In other words, if we can represent each point **z**_*i*_ as a linear combination of all points, matrix *C* will be a matrix of linear coefficients. Consider SVD decomposition of *Z*:


(2) $$\begin{array}{l} Z=U\Lambda V^{T} \end{array}$$

Assuming the number of points *n* is greater than dimension of latent space *q* and matrix *Z* has rank *r*, we can discard all columns of *U* and *V* which are multiplied by zeros. The resulting decomposition is sometimes called “skinny” SVD decomposition:


(3) $$\begin{array}{l} Z=Û\hat{\Lambda }\hat{V} \end{array}$$

Where Û is *q* × *r* matrix, $\hat{\Lambda }$ is *r* × *r* matrix and $\hat{V}$ is *n* × *r* matrix. Notice that *V*^*T*^*V* = *U*^*T*^*U* = *I*, but *VV*^*T*^ ≠ *I*. Then matrix $Q=\hat{V}{\hat{V}}^{T}$ is a self-representation matrix:


(4) $$\begin{array}{l} Z\hat{V}{\hat{V}}^{T}=Û\hat{\Lambda }{\hat{V}}^{T}\hat{V}{\hat{V}}^{T}=Û\hat{\Lambda }{\hat{V}}^{T}=Z \end{array}$$

*The Subspace Separation Theorem* formulated in Kanatani (2001) states the following:

** Theorem 2**. If the αth and βth points belong to different subspaces, then *Q*_αβ_ = 0.

Since Q is a self-representation matrix, *Q*_αβ_ = 0 means that αth point is not necessary in representation of βth point as a liner combination of all points. We can interpret *Q* as an adjacency matrix of a graph with nonzero entries indicating edges. Our intuition is that a collection of points belonging to the same linear subspace can be expressed as a linear combination of one another. Then we can apply a well-known spectral clustering algorithm based on SVD decomposition of a Laplacian of *Q* to find desired labels.

#### 2.3.2. ϵ_*i*_ ≠ 0

There are many techniques to approach fluctuations from an ideal case. For example, we can formulate the noiseless scenario as an optimization problem with exact constraints, and then relax them. Matrix $Q=\hat{V}{\hat{V}}^{T}$ is a solution to the following problem:


(5) $$\begin{array}{l} \underset{C}{\min}rank\left(C\right)\;\text{s.t.}\;Z=ZC \end{array}$$

Indeed, *rank*(*C*) ≥ *rank*(*ZC*) and *Z* = *ZC*, thus *rank*(*C*) ≥ *rank*(*Z*). We also know that $rank\left(\hat{V}{\hat{V}}^{T}\right)=rank\left(Z\right)$ by construction. Therefore, matrix *Q* defined above is a solution to the problem. However, it is not unique. In Liu et al. (2013), the authors show that this is also the solution to a convex analogue of rank minimization problem:


(6) $$\begin{array}{l} \underset{C}{\min}||C||{}_{*}\;\text{s.t.}\;Z=ZC \end{array}$$

Where ||*C*||_*_ is a nuclear norm of matrix *C*. Since this variation of the optimization objective is convex, the solution is unique. This is the formulation of the optimization objective in which, as proposed in Vidal and Favaro (2014), we can relax the constraints:


(7) $$\begin{array}{l} \underset{C}{\min}||C{||}_{*}+\frac{1}{\tau }||Z-ZC{||}_{F}^{2}\;\text{s.t.}\;C=C^{T} \end{array}$$

With τ being some hyperparameter. In this formulation we are allowed to search for a matrix *C* that is not exactly a self-representation matrix, but is close to it within some margin. The authors of this formulation also prove that the minimizer to (7) is unique and can be found in closed form. The optimal solution Q^*^ is given by the following formula:


(8) $$\begin{array}{l} Q^{*}=\hat{V}P\left(\hat{\Lambda }\right){\hat{V}}^{T} \end{array}$$

Where an operator *P* acts on diagonal entries of $\hat{\Lambda }$ as


(9) $$\begin{array}{l} P\left(x\right)=\{\begin{array}{l} 1-\frac{1}{\tau x^{2}}\;x>\frac{1}{\sqrt{\tau }}\\ 0\;x\leq \frac{1}{\sqrt{\tau }} \end{array} \end{array}$$

The topic of subspace clustering is full of intricacies. The number of proposed approaches is growing every year. With this background, we have barely scratched the surface and have covered only those formulas that we utilized in our work.

### 2.4. Affine subspace clustering

The assumption that subspaces are linear is too restrictive since it demands that these subspaces pass through the origin. On the other hand, affine subspaces are allowed to have bias. Instead of expressing latent representations of observed samples as linear combinations of one another, they are expressed as an affine combination and they solve the same problem of finding a sparse representation of a coefficient matrix. Affine combination differs from a linear combination by an additional restriction to linear coefficients: they must sum to one. So, the problem (5) will be rewritten as


(10) $$\begin{array}{l} \underset{C}{\min}rank\left(C\right)\;\text{s.t.}\;Z=ZC,1^{T}C=1^{T} \end{array}$$

In fact, as discussed in Tsakiris and Vidal (2018), we can combine both restrictions in this optimization problem into one by constructing the following matrix


(11) $$\begin{array}{l} \tilde{Z}=\left[\begin{array}{cccc} {\text{z}}_{1} & {\text{z}}_{2} & \ldots & {\text{z}}_{n}\\ 1 & 1 & \ldots & 1 \end{array}\right] \end{array}$$

This is a simple switch to homogeneous coordinates of **z**. Then, problem (10) will have exactly the same form as problem (5):


(12) $$\begin{array}{l} \underset{C}{\min}rank\left(C\right)\;\text{s.t.}\;\tilde{Z}=\tilde{Z}C \end{array}$$

Everything discussed for problem (5) also applies in this case.

## 3. Problem statement

We define a single motion **o** as a sequence of sensor and motor pairs **o** = {(**s**_*t*_, **m**_*t*_)} of some fixed length *T*. Given a set of observed motions {**o**_*i*_} indexed by *i*, find low-dimensional latent vectors {**z**_*i*_} together with generative decoder *d* such that *d*(**z**_*i*_) = **o**_*i*_ for all *i*. Moreover, it should be possible to find motion clusters by analyzing latent vectors {**z**_*i*_} in an unsupervised manner.

## 4. Main results

To solve the given problem, we employed MTRNN architecture with second-order vertical connections and parametric bias.

### 4.1. RNN architecture

**Figure 1:**
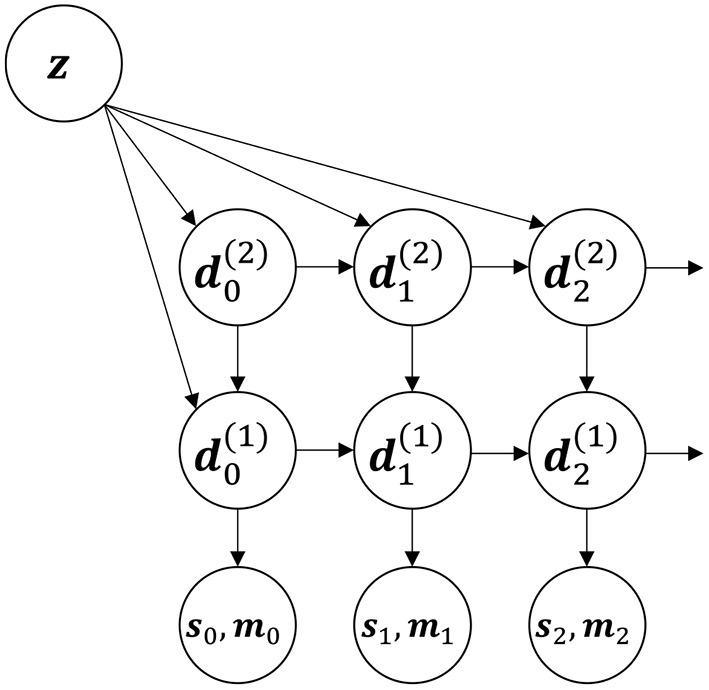
The first three steps of an unfolded recurrent schematic of the RNN architecture used in this paper. There are two layers ${\text{d}}_{t}^{\left(1\right)}$ and ${\text{d}}_{t}^{\left(2\right)}$ in this illustration.

The base model is a variation of MTRNN architecture (see Figure 1). The state of the entire system at time *t* is encoded in a group of vectors $\left\{{\text{d}}_{t}^{\left(j\right)}\right\}$ parameterized by *j*. Each vector ${\text{d}}_{t}^{\left(j\right)}$ together with its update function is referred as the *j*th (dynamic) layer. At every timestep, each vector is updated according to the following formula:


(13) $$\begin{array}{l} \begin{array}{l} {\text{h}}_{t}^{\left(i\right)}=\left(1-\frac{1}{\tau }\right){\text{d}}_{t-1}^{\left(i\right)}\\ \;+\frac{1}{\tau }\left(W^{\left(i\right)}{\text{d}}_{t-1}^{\left(i\right)}+U^{\left(i\right)}\text{u}+{\text{u}}^{T}{\text{A}}^{\left(i\right)}{\text{d}}_{t-1}^{\left(i\right)}+{\text{b}}^{\left(i\right)}\right) \end{array} \end{array}$$

(14) $$\begin{array}{l} {\text{d}}_{t}^{\left(i\right)}=LN\left({\text{h}}_{t}^{\left(i\right)}\right) \end{array}$$

Where τ is a timescale factor for a layer. Typically layers closer to the output have τ closer to one and are called *fast layers*. Conversely, layers farther away from the output have higher τ and are called *slow layers*. The idea is that the fast layers encode more specific details about the current moment of the motion and slow layers encode more abstract information that doesn't change as rapidly.

Next, **u** is equal to a vector from the higher layer ${\text{d}}_{t}^{\left(i+1\right)}$ for all layers, but the very last. For the last layer **u** is equal to *p*(**z**), where *p* is some non-linear function (typically a multi-layered perceptron). In such a way **z** serves the same function for the last layer as any layer for the layer immediately below. Combining that with the fact that higher layers change more slowly in time, we can interpret *p*(**z**) as an infinitely slow layer. This allows us to expand the model in the future and to introduce dynamics to **z**, perhaps combining motions one after another.

*W*^(*i*)^ and *U*^(*i*)^ are trainable weight matrices. **A**^(*i*)^ is trainable third-order tensor for a multiplicative combination of values of the current and a higher layer. It works as a function of two vectors which is linear in both arguments. In literature this is known as a second-order connection (Goudreau et al., 1994), it provides more expressive power compared to ordinary first-order connections at the cost of increased number of trainable parameters. **b**^(*i*)^ is also trainable bias vector.

*LN* in (14) is layer normalization.

Every timestep *t* the output (**s**_*t*_, **m**_*t*_) is generated as a non-linear transformation of the fastest layer ${\text{d}}_{t}^{\left(1\right)}$. To generate the motor command **m**_*t*_ a simple multi-layer perceptron is used. The sensory output **s**_*t*_ is an image in our experiments. To generate it, we used a couple of deconvolution layers with non-linear activation function.

Dynamics of the model match the Equations (1) that are presented in the background section.

Let θ be the vector of trainable parameters of all models. Denote the entire output of the model during the first *T* timesteps as *d*(θ, **z**). Then the loss function will be


(15) $$\begin{array}{l} L\left(\theta ,{\text{z}}_{1},{\text{z}}_{2},...,{\text{z}}_{n}\right)=\overset{n}{\underset{i=1}{\sum }}||d\left(\theta ,{\text{z}}_{i}\right)-{\text{o}}_{i}{||}^{2} \end{array}$$

It is minimized with the usual batch gradient descent. The important thing to notice is that latent encoding vectors **z**_*i*_ are also trainable parameters that we need to optimize, and if initialization of RNN weights is a well-studied topic, initialization of **z**_*i*_ requires some investigation.

### 4.2. Initialization of latent vectors

There are two standard approaches to initialize latent variables: (i) set **z**_*i*_ randomly (e.g., according to a standard gaussian distribution) and (ii) set **z**_*i*_ = **0**.

Random initialization is detrimental for clustering. It doesn't provide any guarantee of structure of latent space. First of all, motions corresponding to different primitives may be located very close to each other in trajectory space; hence, there is no guarantee of distance-based clustering results in latent space. Then, manifolds corresponding to motion primitives are close to affine, according to our main assumption, in trajectory space. But there is no assurance that projection of these manifolds to latent space after learning with random initialization will remain close to affine. Non-linear manifold clustering is a far more complicated problem.

Zero initialization leads to another problem. Because of the batch nature of the optimization algorithm, every gradient step updates the entire θ, but only a fraction of {**z**_*i*_}. These irregularities in latent updates lead to faster convergence of θ compared to **z**_*i*_. For reconstruction of the observed data, it is sufficient for **z**_*i*_ to be distinct enough. Hence, it is not expected that **z**_*i*_ will deviate far from the initial point. If the spread of latent vectors is close to zero, it is too unstable for clustering.

We propose a novel way to initialize. Using the results of theorem 1, we can guarantee that a random linear projection of the entire set of all observed motor sequences **m**_1:*T*_ into **z** will be one-to-one:


(16) $$\begin{array}{l} \text{z}=P{\text{m}}_{1:T} \end{array}$$

Where **m**_1:*T*_ is a sequence of motor commands flattened into a single vector. *P* is a random matrix of compatible dimension with $P_{ij}\sim N\left(0,1/q\right)$, where *q* is the dimension of **z**. With sufficiently expressive generative model architecture it is possible to reconstruct these motor sequences based on the projections exactly:


(17) $$\begin{array}{l} \exists \theta :{\text{m}}_{1:T}=d_{m}\left(\theta ,\text{z}\right) \end{array}$$

For a sensory modality **s**_1:*T*_, it is highly correlated with motor modality; hence, it requires minimal correction to **z**_*i*_ to encode it properly.

Moreover, under the assumption of motion primitives being close to affine subspaces in trajectory space, the projected primitives are also close to affine subspaces in latent space if latent dimensions suffice. Detailed analysis of required dimensions to preserve subspace clusters is given in Li and Gu (2018). The expected scalar product between any two projected vectors *P***a** and *P***b** is very close to the original product **a**·**b**. More formally, the following expressions hold:


(18) $$\begin{array}{l} {𝔼}_{P}\left[P\text{a}\cdot P\text{b}\right]=\text{a}\cdot \text{b} \end{array}$$

(19) $$\begin{array}{l} Var_{P}\left[P\text{a}\cdot P\text{b}\right]\leq \frac{3||\text{a}{||}^{2}||\text{b}{||}^{2}-{\left(\text{a}\cdot \text{b}\right)}^{2}}{q} \end{array}$$

See Appendix A for derivation. From this result it looks as if map *P* is almost isometric for decent values of *q*. This is not true since we are projecting down, but there is a link with *restricted isometry* in cases in which we have a finite number of points. More details are provided by *Johnson-Lindenstrauss lemma* (Johnson and Lindenstrauss, 1984). Unfortunately, this lemma requires *q* to be much higher than we need for efficient encoding, but isometry is not strictly required, since a portion of topological information is also contained in the non-linear decoder *d*. Equations (18) and (19) simply guarantee robustness of a random projection. Directions close to orthogonal in trajectory space will most likely stay close to orthogonal after the projection, even for not so high a *q* compared to that required in the Johnson-Lindenstrauss lemma.

The only assumption we must make is that correction to latent encodings **z**_*i*_ to accommodate sensory information do not disrupt linearity.

## 5. Experimental results

We prepare two experiments to test the hypothesis stated in Section 1. The first is to apply a subspace clustering algorithm for joint angle sequences of robotic arms generated in a demonstration by a human *via* motion capture. The second is to do the same with artificially generated data for a robotic manipulator interacting with an object, and since this data is much more plentiful, to train the RNN generative model with a training/test split of the data and then to apply a subspace clustering algorithm for learned latent encodings. Furthermore, in the second experiment we compare quality of *intra-*primitive generalization for different modes of initialization of latent variables, as well as *inter-*primitive generalization. Generalization is the ability of a trained model to encode new samples with little or no change to its parameters. Intra-primitive generalization is about encoding samples belonging to known primitives. Inter-primitive generalization is the capability to encode entirely new primitives.

### 5.1. Motion capture data

In this experiment we used a Torobo humanoid robot to generate motion trajectories. The torso and head are fixed, leaving only 12 joint angles to control positions of the two arms. We used a motion capture device to manually control the robot arms. The robot is interacting with a red cylindrical object in front of it. There are four motion patterns: (i) touch the top of the object with the left hand, (ii) touch the object on top with the right hand, (iii) touch the object from the left with the left hand, and (iv) touch the object from the right with the right hand. Each pattern is recorded 10 times with the object located in different positions.

Recorded motions naturally vary in length. In order to align them to a single number *T* of discrete timesteps, we used a frequency domain zero padding technique. Having a sequence **m**_1:_*T*__0__ of joint angles with *T*_0_ < *T*, compute the following:


(20) $$\begin{array}{l} {\text{f}}_{1:T_{0}}=F\left({\text{m}}_{1:T_{0}}\right) \end{array}$$

(21) $$\begin{array}{l} {\tilde{\text{f}}}_{1:T}=\left[{\text{f}}_{1:T_{0}/2},\underset{T-T_{0}}{\underset{︸}{0,0,\ldots ,0}},{\text{f}}_{T_{0}/2:T_{0}}\right] \end{array}$$

(22) $$\begin{array}{l} {\tilde{\text{m}}}_{1:T}=F^{-1}\left({\tilde{\text{f}}}_{1:T}\right) \end{array}$$

in which Discrete Fourier Transform F is applied to each joint angle sequence in **m**_1:_*T*__0__ individually. The resulting motion ${\tilde{\text{m}}}_{1:T}$ will have the same “shape” as **m**_1:_*T*__0__, but will have *T* timesteps.

Furthermore, for each joint angle, all values in all sequences were normalized to be in a [−1, 1] interval. This was done to make the contribution of each joint equally important for the trajectories clustering.

**Figure 2:**
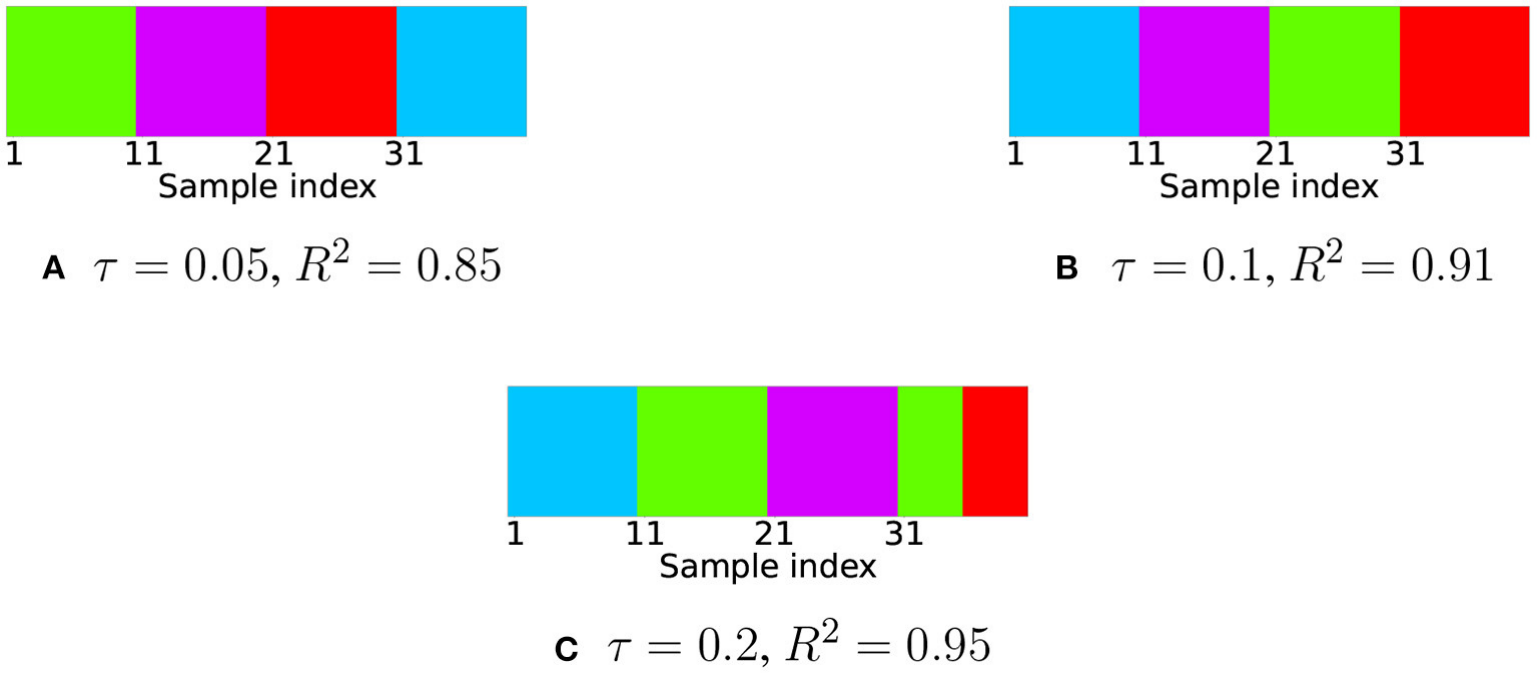
Clustering results for robotic trajectory data acquired by motion capture. Different colors indicate different labels. There are four ground truth clusters corresponding to sample indices 1–10, 11–20, 21–30, and 31–40. In cases **(A,B)** all labels identified correctly in the case **(C)** samples belonging to three out of four primitives are labeled correctly.

We computed the expression (8) for acquired trajectory data with different values of τ. The choice of τ was made based on the coefficient of determination *R*^2^ for reconstruction of trajectories based on the estimated self-expression matrix Q^*^. For 0.85 < *R*^2^ < 0.95 the corresponding values of τ are 0.05 < τ < 0.2. Then, using a spectral clustering algorithm on the obtained matrix Q^*^ we put labels on each sample (see Figure 2).

The result shows that the subspace clustering algorithm is able to correctly assign labels for different motion patterns in the case of very noisy human-made data, which confirms our hypothesis about the close-to-linear distribution of points belonging to the same motion primitive in trajectory space. Note when the value of *R*^2^ is the largest, there are wrong labels after clustering. The reason is clusters are not tightly bounded to corresponding affine subspaces, the exact self-expression matrix is rather far from sparse, so optimization constraints are need to be relaxed. This is due to noise in the data.

### 5.2. Artificial data

**Figure 3:**
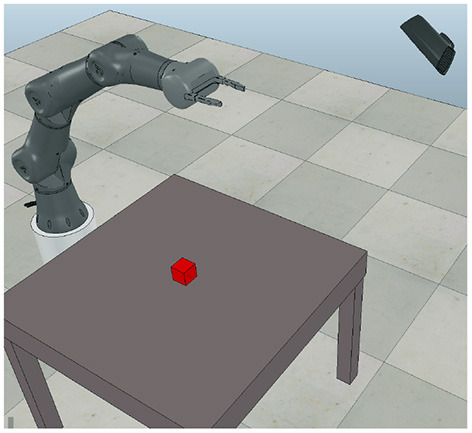
The experimental setup includes a robotic manipulator, a red block on the table and a camera capturing RGB images.

In the second experiment we used a CoppeliaSim simulator (Rohmer et al., 2013) to generate a wide range of trajectories for the Torobo Arm manipulator. The Torobo Arm manipulator position is encoded with seven joint angle positions and two finger positions. The experimental setup consists of the manipulator, a table in front of it, an object with which it interacts, and a camera to record RGB images at every timestep (see Figure 3).

We manually scripted the movement of the tip of the manipulator. All trajectories are acquired by the inverse kinematics solver built into the simulator. Values of joint angles across all motions are normalized so as to be bounded by the interval [−1, 1]. There are seven motion primitives. Each includes 245 motions for different positions of the block, resulting in 1,715 samples. All motion primitives start with the manipulator approaching the block. Then the behavior for each primitive is the following:

- Touch the block on top and stop.
- Grasp the block with two fingers and stop.
- Push the block to the farthest side of the table.
- Pull the block to the closest side of the table.
- Repeatedly touch the block from the right.
- Make a circular motion around the block in the clockwise direction.
- Make a circular motion around the block in the counter-clockwise direction.

Within each primitive the difference between specific motions is only the position of the block on the table and size of the block. The robotic arm always starts from the same position. So in trajectory space, points belonging to each primitive form three-dimensional manifolds.

Every motion takes one minute of real time and is divided into 61 timesteps. At each timestep *t*, beside motor information, we also record an RGB image **s**_*t*_ of size 48 × 64 pixels. The goal is to assign a latent vector **z** to every pair of sequences (**m**_1:*T*_, **s**_1:*T*_), and to build a decoder *d* such that *d*(θ, **z**) = (**m**_1:*T*_, **s**_1:*T*_). The decoder is an RNN model from the previous section. We used two dynamic layers, 32 variables in the fast layer and 12 in the slow layer. We test three ways to initialize latent variables **z** packed into a single matrix *Z*, described in the previous section: (i) zero initialization, (ii) random initialization, and (iii) random linear projection of the motor trajectory data.

#### 5.2.1. Intra-primitive generalization

To evaluate the model we split the entire dataset into two parts: one is used to train both **z** for each sample and model parameters θ, and the other is to train **z** with parameters θ fixed. We refer to these parts as the “training dataset” and the “evaluation dataset.” Only 20% of the entire dataset is randomly assigned to the training part, and the rest is for evaluation. Samples of all primitives are present in both training and evaluation datasets, so we indeed test intra-primitive generalization.

**Figure 4:**
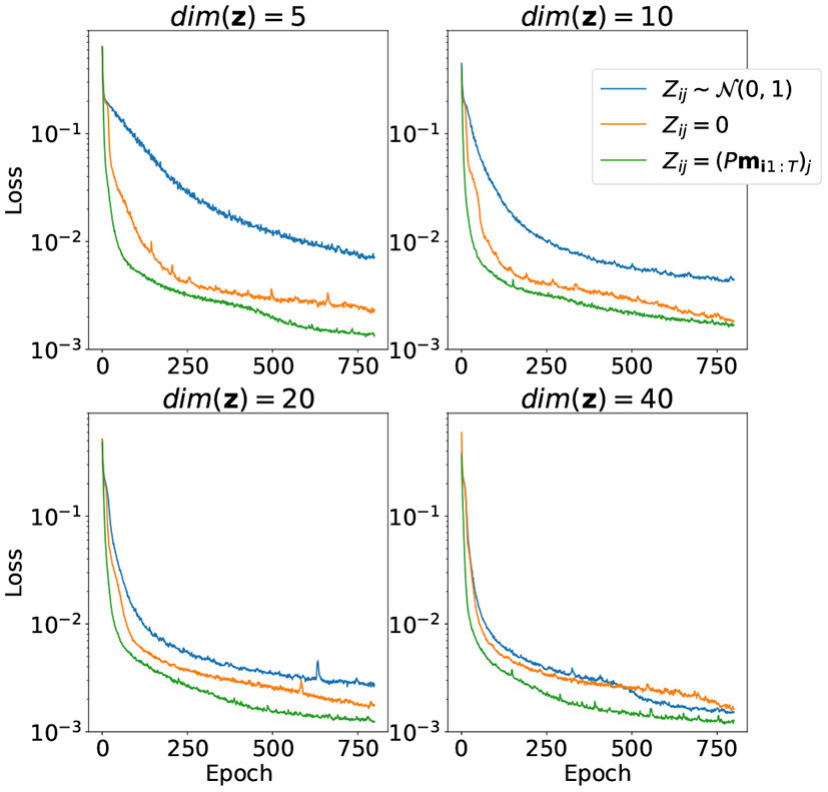
Convergence of the loss function over 800 training epochs for different ways to initialize matrix *Z* and different dimensions of **z**. Both model parameters θ and matrix *Z* are trained.

The results of training the model parameters θ together with latent variables **z** for the training part of the samples for different dimensions of latent space depicted in Figure 4. Random initialization performs very badly for low-dimensional latent space. Zero initialization, on the other hand, differs very slightly from random the linear projection case.

**Figure 5:**
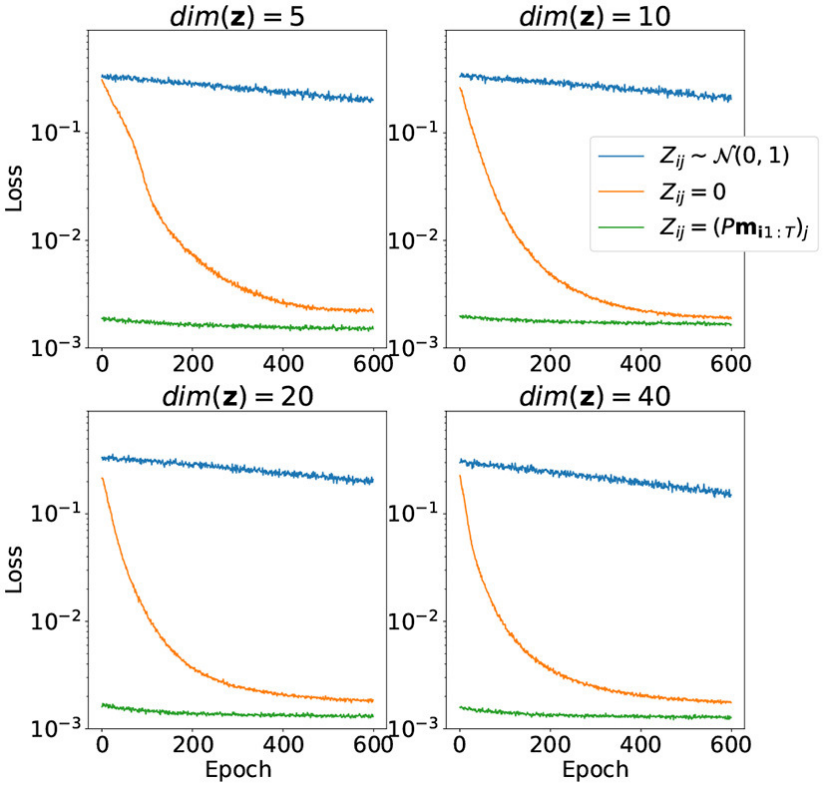
Convergence of the loss function over 600 training epochs for different ways to initialize matrix *Z* and different dimensions of **z**. Only matrix *Z* is trained, model parameters θ are fixed.

The results of training only the latent variables **z** for the evaluation part of the dataset with model parameters with fixed θ are depicted in Figure 5. Notice that the case with random initialization did not converge at all. This means that the model trained this way has no generalization capacity and is incapable of representing trajectories it did not see during training. On the other hand, initialization with random linear projection yields low loss values on evaluation stage even before adjustments of latent variables *via* training, which means that the initial distribution of projected points did not change much during the first part of training. It was good from the start, and even addition of visual information to the loss function didn't affect the result very much.

This may resemble the resulting performance of trained models for zero latent initialization and random projection latent initialization, which are very similar, but some primitives from the experimental setup require more precision than others. In particular, pushing and pulling the block as well as grasping it can fail easily if the gripper fingers miss just a bit. The success rate of these tasks determined by qualitative assessment of recorded video is presented in Table 1.

**Table 1:** Success rates of trained models for sensitive primitives.

**Primitive**	***Z*_*ij*_ = 0**	***Z*_*ij*_ = (***m*_i1:*T*_)_*j*_****
Pull the block	0.86	0.92
Push the block	0.9	0.94
Grasp the block	0.8	1.0

Resulting generated joint angles and image sequences are very close to ground truth for all methods. Refer to Appendix B to see the comparison of generated motor and sensory data with ground truth.

Initial positions of the block on the table. There are five different possible sizes of the block at each position denoted by overlapping squares.

**Figure 6:**
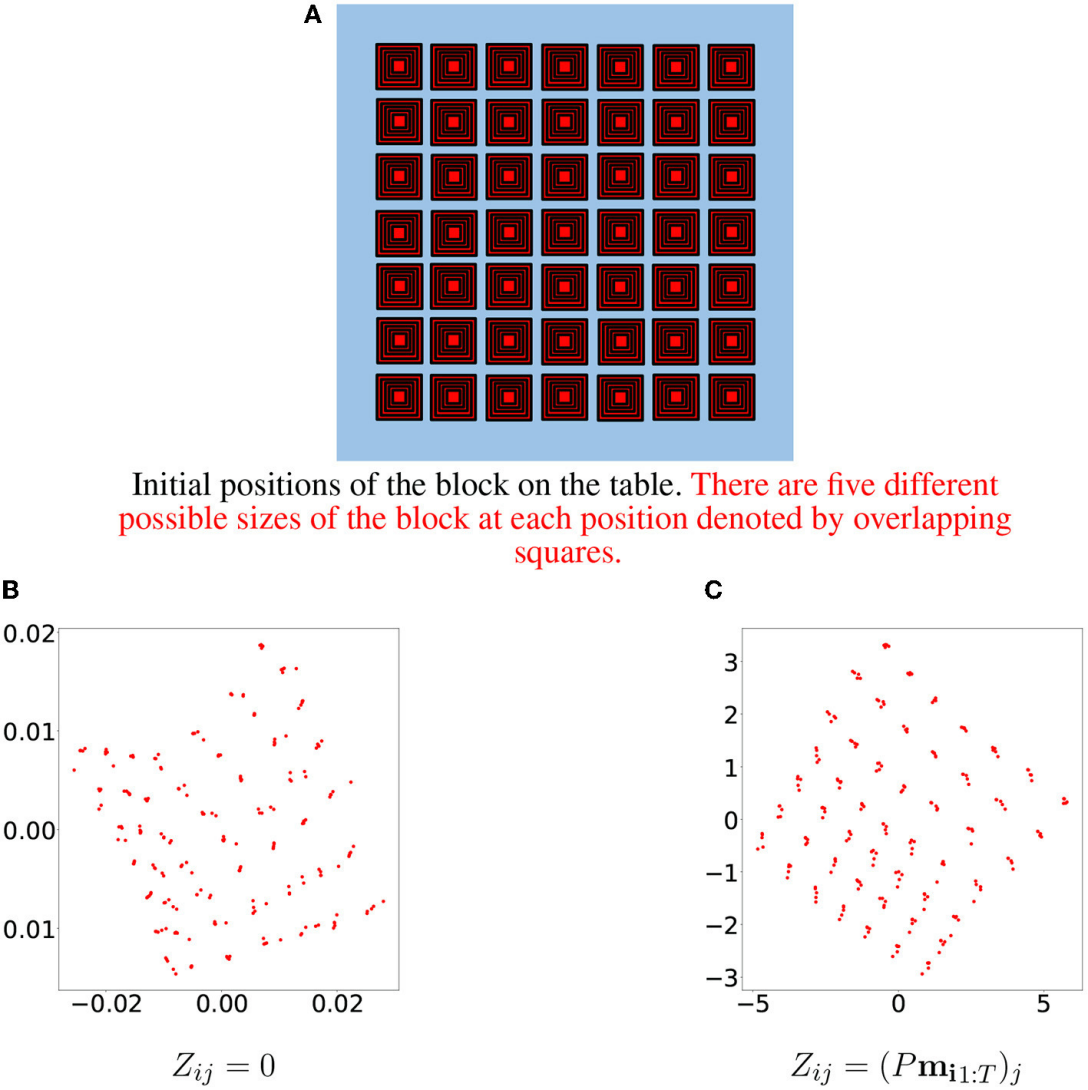
Initial positions of the block are depicted in **(A)**. Each position corresponds to five samples differing in block size. The first two principal components of the learned latent vectors for samples belonging to one specific primitive are depicted in **(B)** for the zero initialization method and in **(C)** for the random linear projections initialization method.

By examining resulting latent vectors corresponding to samples from one primitive, we can see that the random linear projection initialization method yields a more “structured” result (see Figure 6). A 3D visualization of the three principal components of latent vectors corresponding to samples of three primitives is available at the following link: https://doi.org/10.6084/m9.figshare.19235034.v2.

#### 5.2.2. Affine subspace clustering of latent space

**Figure 7:**
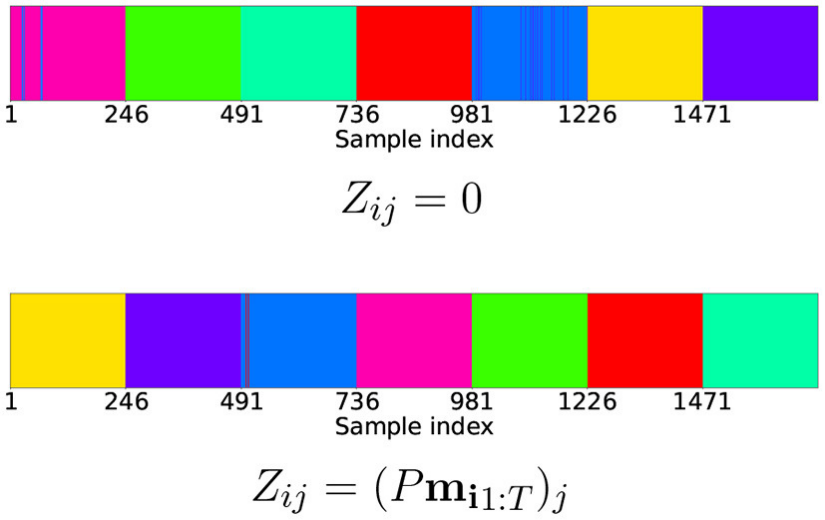
Clustering results for latent encodings for different initial values of *Z*. All 1715 samples are presented in order of consecutive 245-element batches, in which every batch is a collection of motions belonging to the same primitive. The coefficient τ is set in such a way, that in both cases, the coefficient of determination *R*^2^ = 0.9999. This means resulting manifolds are very close to affine.

Next, to compare affine subspace clustering results, we won't even consider the case of random initialization, since it does not correctly encode the evaluation part of the dataset. We use algorithms discussed in the background section for matrix *Z* to predict cluster labels for the data obtained by two steps of training and evaluation. A comparison of the two methods to initialize *Z* is depicted in Figure 7. Both cases are for *dim*(**z**) = 40. They yield similar results, meaning that even without presenting motion information for initial values of latent variables, they self-organize in a union of close to affine manifolds. This shows that initial values of latent variables obtained by proposed random linear projection already have some properties of fully trained latent representation.

#### 5.2.3. Inter-primitive generalization

To test inter-primitive generalization ability we perform seven independent tests for each primitive with different splits of the whole dataset into two parts: the first part contains samples of six primitives for initial training of the model and the second part contains samples of the remaining primitive. We also add a small portion (10%) of samples from the first part to the second part to avoid forgetting. Incremental learning is a difficult topic and we won't discuss its intricacies here, since it is preferable to use the simple solution described above. Both model parameters θ and latent encodings are trained in both parts, but the two parts are trained consecutively.

**Figure 8:**
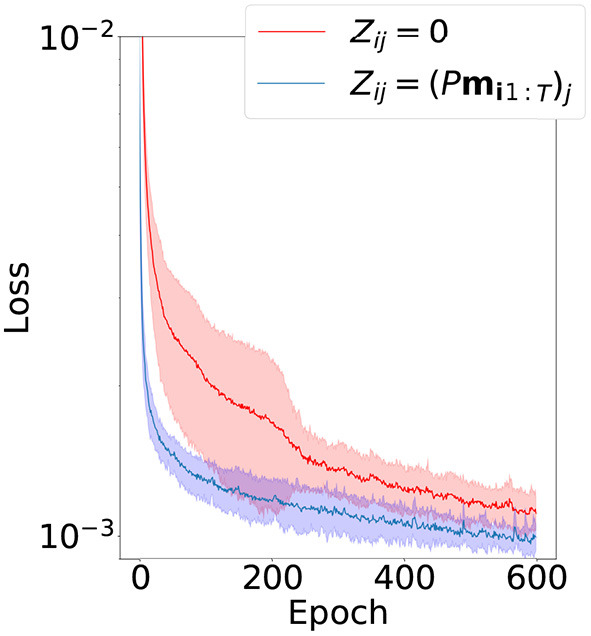
Mean and standard deviation of the convergence of the loss function in the second part of inter-primitive generalization experiment. The dimension of latent vectors is 20.

Since this problem is considerably more complex than intra-primitive generalization, we compare only two successful latent variable initialization methods from the previous experiment: zero initialization and proposed random linear projection. Results of training the second part are depicted in Figure 8. The proposed method shows slightly improved results in terms of convergence speed, final value of the loss function, and robustness of the learning.

#### 5.2.4. Affine subspace clustering for extended dataset

**Figure 9:**
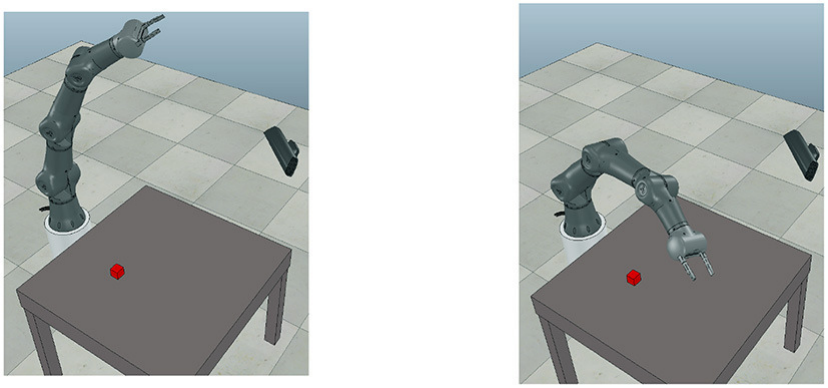
Examples of different initial positions of Torobo Arm in extended dataset.

We extended the artificial dataset with trajectories corresponding to different initial positions of the arm to increase variety of motions within primitives. See example of new initial positions at Figure 9. In total there are three new degrees of freedom corresponding to different values of initial joint angles. There are 675 motions for each primitive.

**Figure 10:**
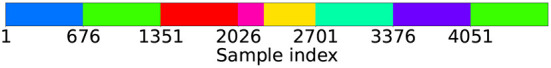
Clustering results for entire trajectories in extended dataset. All 4,725 samples are presented in order of consecutive 675-element batches, in which every batch is a collection of motions belonging to the same primitive.

Result of subspace clustering for these trajectories is depicted in Figure 10. Notice that second and third “primitives” are clustered together. They correspond to circular motion around the block in clockwise and counter-clockwise directions. And indeed it is hard to tell whether it is one primitive or two different primitives. Nevertheless, the dataset clearly follows the assumptions about structure of trajectories and proposed algorithm will benefit in this case.

## 6. Summary and discussion

In this paper we investigated the structure of robotic motion primitives in trajectory space and ways of efficiently encoding that structure. The distinctive feature of each primitive is that the set of all motions belonging to this primitive lies on a low-dimensional manifold embedded in trajectory space, which was confirmed by experiments in which we were able to reconstruct artificially generated robotic motions from random linear projections of its motor trajectory data using an RNN model. Moreover, these manifolds are close to affine subspaces, which enables us to use a subspace clustering algorithm to label collections of motion in an unsupervised manner. This claim comports with clustering results of data obtained with a motion-capture device. Another assumption is correlation of visual information with motor commands. In our experiments, we showed that only slight correction to initial values of latent variables obtained by random linear projection is required to minimize the combined loss function for motor and visual data. Lastly, to show that random linear projections do not disturb affine subspace clusters of trajectory space, it is still possible to do subspace clustering of projected data for sufficiently large dimensions of latent space. Clustering results for latent encodings show sufficient precision to support this claim. Initialization of the latent variable by random linear projections improves intra- and inter-primitive generalization capabilities, compared to conventional initialization methods.

One crucial limitation of the proposed approach is the assumption about fixed number of timesteps per motion within each primitive. A possible solution is described in the first experiment with motion capture data, sequences within which naturally vary in length. All sequences are interpolated with additional points to have the same number of timesteps. It erases information about speed of motions, but it still can be recovered during the training process.

Well-structured latent spaces benefit generative models for searching appropriate encodings *via* regression. For example, provided perception and initial joint angles position at the first timestep, it is possible to perform regression over latent space to find suitable representation and generate the rest of the sequence, similar to Ito and Tani (2004). Subspace clustering can potentially allow selection of specific primitive to be generated by restricting the search to specific affine subspace.

To model more complex behaviors composed of many consecutive primitives, instead of using a single latent vector **z** of fixed dimension a sequence of such vectors is usually used. In the future, we are planning to extend the latent variable initialization algorithm by random linear projections to a sequence of latent vectors. This can be done *via* one-dimensional convolution through time of a random linear projection with a long motor sequence. There are some already mentioned challenges, such as primitives that might have a different number of timesteps and an indistinct borderline between primitives.

Another point is that visual perception information is not used to initialize encodings. The problem here is that only a small portion of each perceived image is relevant to motion. The rest is background noise that will clutter random projection. Some attention mechanism will potentially alleviate the problem. Vision information is highly correlated with motor commands, but part of it is independent, such as colors of objects with which the robot is interacting.

## Data availability statement

The raw data supporting the conclusions of this article will be made available by the authors, without undue reservation.

## Author contributions

VN contributed to conception and design of the study, experimental design, and proof writing. All authors contributed to manuscript revision, read, and approved the submitted version.

## Conflict of interest

The authors declare that the research was conducted in the absence of any commercial or financial relationships that could be construed as a potential conflict of interest.

## Publisher's note

All claims expressed in this article are solely those of the authors and do not necessarily represent those of their affiliated organizations, or those of the publisher, the editors and the reviewers. Any product that may be evaluated in this article, or claim that may be made by its manufacturer, is not guaranteed or endorsed by the publisher.
