# Supplementary material for: Initialization of latent space coordinates via random linear projections for learning robotic sensory-motor sequences
Source: Front Neurorobot. 2022 Sep 14;16:891031. doi: 10.3389/fnbot.2022.891031 (PMC9515618; doi:10.3389/fnbot.2022.891031)
Supplement: Supplementary file 1 [file Data_Sheet_1.pdf]

APPENDIX A: RANDOM PROJECTION ROBUSTNESS

$\mathbf{a}$, $\mathbf{b} \in \mathbb{R}^k$, P is a matrix of compatible dimension which defines a random linear map from $\mathbb{R}^k$ to $\mathbb{R}^q$, $P_{ij} \sim \mathcal{N}(0, 1/q)$. The derivation of (18) in the main text is straightforward:

$$\mathbb{E}_P[P\mathbf{a} \cdot P\mathbf{b}] = \mathbb{E}_P \left[\sum_{j=1}^{q} \left(\sum_{i=1}^{k} P_{ij} a_i \right) \left(\sum_{i=1}^{k} P_{ij} b_i \right) \right] \tag{1}$$

$$= \mathbb{E}_P \left[\sum_{j=1}^{q} \left(\sum_{i=1}^{k} P_{ij}^2 a_i b_i + \sum_{i=1}^{k} \sum_{l \neq i}^{k} P_{ij} P_{lj} a_i b_l \right) \right] \tag{2}$$

$$= \sum_{j=1}^{q} \left(\sum_{i=1}^{k} \underbrace{\mathbb{E}_P[P_{ij}^2]}_{=1/q} a_i b_i + \sum_{i=1}^{k} \sum_{l \neq i}^{k} \underbrace{\mathbb{E}_P[P_{ij}]}_{=0} \underbrace{\mathbb{E}_P[P_{lj}]}_{=0} a_i b_l \right) \tag{3}$$

$$= \sum_{j=1}^{q} \left(\sum_{i=1}^{k} \frac{1}{q} a_i b_i \right) \tag{4}$$

$$= \frac{1}{q} \sum_{j=1}^{q} \left(\sum_{i=1}^{k} a_i b_i \right) \tag{5}$$

$$= \mathbf{a} \cdot \mathbf{b} \tag{6}$$

jth component of vector $P\mathbf{a}$ is a normally distributed random variable:

$$(P\mathbf{a})_j = \sum_{i=1}^{k} P_{ij} a_i \sim \mathcal{N}\left(0, \frac{\sum_{i=1}^{k} a_i^2}{q}\right) = \mathcal{N}\left(0, \frac{\|\mathbf{a}\|^2}{q}\right) \tag{7}$$

Same applies for $(P\mathbf{b})_j$. We use this fact in the following derivation:

$$Var_P[P\mathbf{a} \cdot P\mathbf{b}] = \sum_{j=1}^{q} Var_P \left[\left(\sum_{i=1}^{k} P_{ij} a_i \right) \left(\sum_{i=1}^{k} P_{ij} b_i \right) \right] \tag{8}$$

$$= \sum_{j=1}^{q} \mathbb{E}_P \left[\left(\sum_{i=1}^{k} P_{ij} a_i \right)^2 \left(\sum_{i=1}^{k} P_{ij} b_i \right)^2 \right] - \sum_{j=1}^{q} \underbrace{\mathbb{E}_P \left[\left(\sum_{i=1}^{k} P_{ij} a_i \right) \left(\sum_{i=1}^{k} P_{ij} b_i \right) \right]^2}_{=(\mathbf{a}\cdot\mathbf{b})^2/q^2, \text{from (1-5)}}$$

$$= \sum_{j=1}^{q} \mathbb{E}_P \left[\left(\sum_{i=1}^{k} P_{ij} a_i \right)^2 \left(\sum_{i=1}^{k} P_{ij} b_i \right)^2 \right] - \sum_{j=1}^{q} \frac{(\mathbf{a} \cdot \mathbf{b})^2}{q^2} \tag{9}$$

$$\leq \sum_{j=1}^{q} \sqrt{ \mathbb{E}_P \left[\left(\sum_{i=1}^{k} P_{ij} a_i \right)^4 \right] \mathbb{E}_P \left[\left(\sum_{i=1}^{k} P_{ij} b_i \right)^4 \right] } - \frac{(\mathbf{a} \cdot \mathbf{b})^2}{q}$$

$$= \sum_{j=1}^{q} \sqrt{ \frac{3\|\mathbf{a}\|^4}{q^2} \cdot \frac{3\|\mathbf{b}\|^4}{q^2} } - \frac{(\mathbf{a} \cdot \mathbf{b})^2}{q} \tag{10}$$

$$= \sum_{j=1}^{q} \frac{3\|\mathbf{a}\|^2 \|\mathbf{b}\|^2}{q^2} - \frac{(\mathbf{a} \cdot \mathbf{b})^2}{q} \tag{11}$$

$$= \frac{3\|\mathbf{a}\|^2 \|\mathbf{b}\|^2 - (\mathbf{a} \cdot \mathbf{b})^2}{q} \tag{12}$$

We used Cauchy–Schwarz inequality in (9-10). In (10) under the square root there is a product of the 4th moments of two normal distributions derived in (7).

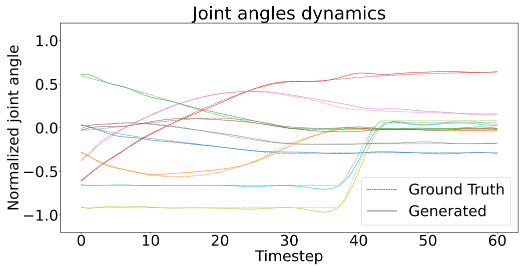

”Grasp the block” primitive sample

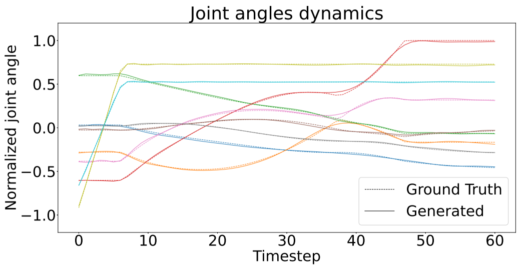

”Push the block” primitive sample

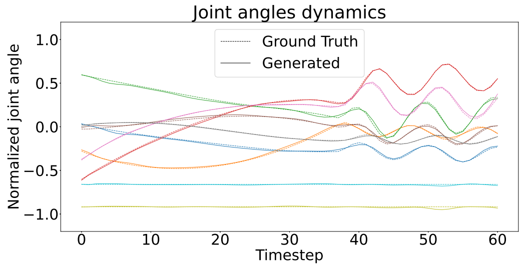

”Circular motion around the block” primitive sample

Figure 1. Sequences of joint angles for seven joints and two fingers of the robotic arm generated by model and the ground truth. All models with sufficiently low loss function values show similar results. This image is for the model with latent dimension equal 40 and random linear projection initialization method.

APPENDIX B: COMPARISON OF GENERATED DATA WITH GROUND TRUTH

Comparison of generated sequences of joint angles with ground truth is presented in Fig. 1. Examples of recorded image sequence together with prediction is available at the following link: https://doi.org/10.6084/m9.figshare.19235277.v2

Table 1. Recurrent connection parameters

Parameter Tensor	Shape
$W^{(1)}$	$(32, 32)$
$U^{(1)}$	$(12, 32)$
$A^{(1)}$	$(12, 32, 32)$
$b^{(1)}$	(32)
$W^{(2)}$	$(12, 12)$
$U^{(2)}$	$(dim(\mathbf{z}), 12)$
$A^{(2)}$	$(dim(\mathbf{z}), 12, 12)$
$b^{(2)}$	(12)

Table 2. Transposed convolutional layers parameters

Layer	Kernel Shape	Stride
$TC^{(1)}$	$(8, 10, 256, 128)$	$(2, 2)$
$TC^{(2)}$	$(2, 2, 128, 64)$	$(2, 2)$
$TC^{(3)}$	$(2, 2, 64, 3)$	$(2, 2)$

APPENDIX C: RNN ARCHITECTURE

Exact shapes of parameter tensors for the recurrent part of the model used in experiments are presented in table 1. Here layer 1 is the fast layer and layer 2 is the slow layer. Motor output at each timestep is a single layer without activation. Image output is a sequence of one full-connected layer and three transposed convolutional layers $\{TC^{(1)}, TC^{(2)}, TC^{(3)}\}$ with $tanh$ activation in between. See table 2 for exact kernel shapes. Kernel shape is presented in the following format: (height, width, input channels, output channels).

For optimization of the parameters we used Adam optimizer (Kingma and Ba, 2015) with $\alpha = 1e - 3$, $\beta_1 = 0.9$ and $\beta_2 = 0.999$.

Weights are initialized with a simple uniform random distribution.

REFERENCES

Kingma, D. P. and Ba, J. (2015). Adam: A method for stochastic optimization. *CoRR* abs/1412.6980
